# Colour Terms Affect Detection of Colour and Colour-Associated Objects Suppressed from Visual Awareness

**DOI:** 10.1371/journal.pone.0152212

**Published:** 2016-03-29

**Authors:** Lewis Forder, Olivia Taylor, Helen Mankin, Ryan B. Scott, Anna Franklin

**Affiliations:** 1 The Sussex Colour Group, School of Psychology, University of Sussex, Falmer, Brighton, United Kingdom; 2 School of Psychology, University of Sussex, Falmer, Brighton, United Kingdom; Centre de Neuroscience Cognitive, FRANCE

## Abstract

The idea that language can affect how we see the world continues to create controversy. A potentially important study in this field has shown that when an object is suppressed from visual awareness using continuous flash suppression (a form of binocular rivalry), detection of the object is differently affected by a preceding word prime depending on whether the prime matches or does not match the object. This may suggest that language can affect early stages of vision. We replicated this paradigm and further investigated whether colour terms likewise influence the detection of colours or colour-associated object images suppressed from visual awareness by continuous flash suppression. This method presents rapidly changing visual noise to one eye while the target stimulus is presented to the other. It has been shown to delay conscious perception of a target for up to several minutes. In Experiment 1 we presented greyscale photos of objects. They were either preceded by a congruent object label, an incongruent label, or white noise. Detection sensitivity (d’) and hit rates were significantly poorer for suppressed objects preceded by an incongruent label compared to a congruent label or noise. In Experiment 2, targets were coloured discs preceded by a colour term. Detection sensitivity was significantly worse for suppressed colour patches preceded by an incongruent colour term as compared to a congruent term or white noise. In Experiment 3 targets were suppressed greyscale object images preceded by an auditory presentation of a colour term. On congruent trials the colour term matched the object’s stereotypical colour and on incongruent trials the colour term mismatched. Detection sensitivity was significantly poorer on incongruent trials than congruent trials. Overall, these findings suggest that colour terms affect awareness of coloured stimuli and colour- associated objects, and provide new evidence for language-perception interaction in the brain.

## Introduction

The idea that language can affect how we see the world continues to create controversy [1–4]. One reason for this is that it is at odds with models of perception that propose the relationship between a stimulus and response is a purely feed-forward process (e.g., [5,6]). If language does affect how we see the world, this suggests a form of interaction between higher-level cognition and lower-level processing systems (e.g., ([7–10]). This debate has substantial implications for our understanding of the way that we perceive and interact with the external world.

There is a growing body of behavioural research that supports the idea that language affects visual perception. For example, people tend to be faster at identifying target letters on a search task when they actively name the letters [11], suggesting that the linguistic activity of verbalising can affect visual identification. The way people make same/different judgements for dot-cross configurations suggests there is a relationship between lexicalized spatial categories (“up”, “left”, etc.) and the perceptual processing of categorical and spatial relations [12]. In this study [12], biases in the perception of spatial relations were found that correlated with patterns of lexicalisation. Further, there appears to be a direct relationship between the way different cultures talk about the world and performance on tasks exploring visual perception. Among other things this has been demonstrated for the way in which people respond to colour. For example, speeded colour discrimination [13] as well as short- and long-term memory recall [14] tends to be better around language-specific colour category boundaries. These data clearly indicate a relationship between language and visual perception. However, a fundamental question here is whether language directly affects activity in the visual processing system in an interactive manner (e.g., [8–10], or whether language instead affects perception after visual processing, in a manner consistent with feed-forward models of perception (e.g., [5,6]).

In the label-feedback hypothesis, Lupyan [3] argues that language has the capacity to affect low-level visual processing. According to this hypothesis, observable differences in behaviour on tasks probing visual perception, such as those reported above [11–14], occur because language exerts a top-down influence on bottom-up neural activity in the visual processing system. Critically, this view holds that language can affect the earliest ‘perceptual’ stages of visual processing. However, an issue here that arises from behavioural data is that it is often difficult to differentiate between whether language genuinely affects activity in the visual system, or whether language instead affects subsequent higher-level ‘post-perceptual’ activity, such as attention, semantic processing or decision making [15,16]. These contrasting views explicitly differ in what they say about the specific timing of the relationship between language and visual processing and consequently question the stage of visual processing that language can penetrate. In other words, does language affect early stages of visual processing [3], or does this effect reside in later post-perceptual processes [15,16]?

Electrophysiological measurements provide precise information about the time course of visual processing activity [17]. As such, they can be used to help clarify the time course of the relationship between language and visual perception. There are several studies in this area that suggest that language does affect activity in early stages of visual processing. Thierry et al. [18] compared colour processing in native Greek speakers and native English speakers by measuring event-related potentials. These languages differentially categorize the colour blue: unlike the English language, the Greek language divides this colour into two distinct colour categories for lighter and darker shades [19]. The authors showed that this linguistic difference in colour categorisation was associated with different patterns of neural activity for these colours as early as 100 ms after a colour was presented. Similar effects were reported in a study of native English speakers in a colour processing task [20] and in an object recognition task [21]. Importantly, at this time point in visual processing (i.e., 100 ms) activity in the visual system is thought to reflect early, lower-level processes [22–24]. Higher-level post-perceptual processes are thought to occur several hundred milliseconds later [25,26].

There is also evidence that language effects activity in areas of the brain known to be specialised for vision. In a colour naming task, functional magnetic resonance imaging (fMRI) revealed that cortical activity in human ventral area V4 and VO1 was modulated when participants actively named the colour of the stimuli [27]. These areas are known to play a specific role in colour processing [28,29]. On one hand this may suggest a direct link between language and visual perception, and therefore strong evidence that language affects low-level activity in the visual processing system. However, there is debate in this area. The effects in V4 and VO1 reported by [27] were found when participants actively named (and therefore attended to the visual stimuli). In contrast, no modulation of neural activity in visual cortex was found when attention was directed away from colour stimuli. Likewise, in two studies in which participants passively attended to colours, no modulation of cortical activity was found in visual cortex [30,31]. This contrasts to prior findings that language affects cognitive processing regardless of whether attention is deployed to a task (for a recent review, see [32]). Likewise, some electrophysiological studies fail to find effects in early stages of visual processing and instead find effects in later post-perceptual processes [33,34].

A different approach to identifying whether language affects activity in early visual processes or later post-perceptual processes was adopted by [35]. Rather than measuring neural activity, these authors deliberately suppressed images from visual awareness using a form of binocular rivalry known as continuous flash suppression (CFS; [36]). Importantly, evidence indicates that CFS disrupts visual processing prior to semantic (i.e. post-perceptual) analysis [37]. This conclusion is based on a number of prior CFS studies which have demonstrated the absence of semantic priming with a variety of stimuli. For example, [38] demonstrated that the conscious perception of a semantically related object failed to influence the perception of a CFS suppressed object. Similarly, [39] demonstrated that the conscious presentation of semantically related words failed to influence the time taken for a CFS suppressed word to break through to conscious awareness. In their study [35], the authors investigated whether verbal labels affected whether participants would eventually perceive the suppressed images compared to when audio noise (the baseline condition) preceded the image. When verbal labels were used they either matched or did not match the image. For example, participants would hear the word “pumpkin” before a suppressed image was displayed, which was either a pumpkin or a different image. It was hypothesised that if language penetrates early stages of visual processing, a verbal label would affect whether participants detected the presence of the suppressed image. Alternatively, if language instead affects post-perceptual mechanisms, verbal labels were predicted to have no effect on detection. The study found that the type of audio cue did affect performance: detection was significantly better for trials where the auditory stimulus was a verbal label congruent with the suppressed image than for those where the stimulus was simply noise. Further, detection was significantly worse on incongruent trials compared to noise trials.

To date one study has replicated this method [40]. These authors explored whether this effect would replicate and whether it occurs more strongly for suppressed images presented to the right visual field compared to the left visual field. They sought to make this comparison because it is known that visual input to the right visual field is projected and processed contralaterally in the left hemisphere, which is known to be more specialised for the processing of language [41,42]. Prior findings suggest that this affects performance on tasks probing visual perception [43,44]. In their study [40], they replicated the effect reported by [35] and showed that the detection of suppressed images is affected by the congruency of a preceding verbal label. However, in contrast to prior studies investigating hemispheric asymmetry, it was found that detection performance to stimuli presented to the left visual field was more greatly influenced by verbal labels, rather than the right visual field. While the implications of this finding for the debate about language and perception remain to be resolved, an issue here is that there is evidence for a right hemispheric dominance in the processing of spatial attention [45,46], which may alternatively account for this finding.

In the study by Lupyan and Ward [35] and the study by Sun et al. [40], language affected the detection of suppressed stimuli with this effect appearing to arise from a top-down effect of language on early perceptual stages of visual processing (e.g., [37]). This is clearly important to the debate about language and perception. If this finding represents a general rule in the influence of language on vision then it should also generalise to other types of label-stimulus associations. The current study aimed to investigate this over the course of three experiments. In Experiment 1 we investigated whether we could replicate this effect, and used greyscale photos of objects. The objects were suppressed from awareness through CFS and were preceded by an audio cue. The cue was either a verbal label that matched or did not match the object, or it was audio noise. On half of the trials there was no object present so as to account for potential response bias concerning detection performance. In Experiment 2 we adopted the same method but instead investigated if the effect would generalise to different stimuli. We used patches of colour and tested whether colour terms influenced their detection. In both of these experiments the verbal labels were direct cues to a suppressed stimulus: in Experiment 1 (object label) it was a direct cue to the spatial configuration of a suppressed stimulus and in Experiment 2 (colour term) it was a direct cue to the colour of the stimulus. In contrast to this, in Experiment 3 we tested the strength of this effect by investigating whether an *indirect* cue to a suppressed stimulus also affected detection. For this we presented suppressed greyscale images of objects preceded by either an auditory colour term or noise, where each colour term either matched or did not match the object’s characteristic colour.

## Experiment 1: Object Targets and Object Labels

Experiment 1 aimed to replicate the finding of [35] using a highly similar method. Using CFS, we suppressed greyscale photos of objects from visual awareness and measured participants’ performance at detecting them. The objects were either preceded by a congruent auditory object label (e.g., hear ‘frog’ before ‘frog’ image), an incongruent object label that did not match the object (e.g., hear ‘frog’ before ‘dog’ image), or audio noise. Noise trials act as a baseline condition for subsequent comparison to congruent and incongruent trials. There were a larger proportion of congruent trials versus incongruent trials so that the object labels were predictive of the hidden objects. On half of all trials there was no hidden object. These object-absent trials were necessary to identify participants, who might have a tendency to report that they saw a suppressed object on a trial when they had not. Further, by combining these performance data with data from object-present trials, we calculated a measure of detection sensitivity (*d’*). This measure is valuable because it takes into account a bias that may exist to respond in a particular way under conditions of uncertainty. Here, data from ‘hits’ and ‘misses’ (the correct and incorrect response on object-present trials respectively) are combined with data from ‘correct rejections’ and ‘false alarms’ (the correct and incorrect response on object-absent trials) to calculate *d’*. By measuring *d’*, as well as hit rates (accuracy) and reaction time, we investigated whether object labels affected participants’ performance for detecting hidden objects, in a manner comparable to [35].

The current study had a few minor differences in the method to [35]. First, we used a mirror stereoscope to present visual noise to one eye and an object to the other eye, rather than red-cyan anaglyph glasses. This was to permit the same method to be applied to colour stimuli in Experiment 2. We also used square random white noise patterns as visual noise to mask the target objects, rather than coloured, curved line segments. These square noise patterns provide a strong image signal and contain a broad distribution of high spatial frequency content. Further, unlike the curved line segments used by [35], they have previously been investigated in a psychophysical paradigm to ensure successful masking [47].

We anticipated that, if the effect reported by [35] is reliable, we would observe a similar pattern of results. Specifically, compared to baseline trials, we predicted that detection would be significantly better in congruent trials and significantly worse in incongruent trials.

### 1.1. Methods

#### 1.1.1. Participants

Twenty British English speakers took part (16 female; mean age = 20.4; *SD* = 0.7; Range = 19–22). Participants were recruited from the University of Sussex. All participants were screened for colour vision deficiencies using the Ishihara test [48] and the City University Test [49] presented under natural daylight. Observers were naive to the purpose of the study, provided written informed consent and their time was reimbursed with money or research credits. The study was approved by the Cluster-based Ethics Research Committee of Psychology and Life Sciences at the University of Sussex.

#### 1.1.2. Stimuli and set up

Participants were seated in a dark room, the only source of light was a 22" Diamond Pro 2070SB CRT monitor (Mitsubishi, Tokyo, Japan; colour resolution: 8 bits∕channel; spatial resolution: 1280 × 1024; refresh rate: 100 Hz) located at a distance of 55 cm. Participants viewed dichoptic stimuli through a mirror stereoscope (NVP3D, La Croix-sur-Lutry, Switzerland) fixed to a chin rest. The background grey was metameric with illuminant C and had a luminance of 40 cd/m^2^. Both dichoptic stimuli were boxes (5.4° × 5.4°) surrounded by a black border (0.4°) with a horizontal gap between the boxes of around 8° (the size of the gap could be varied to suit participants individually). When viewed through the stereoscope each eye viewed a different box but participants perceived there to be a single box (the two boxes fused binocularly). To align both boxes with each participant’s eyes, both boxes could be moved by pressing computer keys. The same four vertical and four horizontal white lines (width: 0.3°) were added to the border of both boxes to assist binocular fusion as well as a fixation cross (1° × 1°) in the centre of both boxes. During trials the dominant eye was presented with a masking stimulus that changed at a rate of 10 Hz. We used a similar approach to generate the masking stimulus as [47], whereby the masking stimulus was a grid of 27 × 27 greyscale squares (each 0.2° × 0.2° and metameric with illuminant C) that filled the entire space inside the border of the box. Each time the masking stimulus changed, the luminance of each greyscale square was randomly selected from 27 possible luminance values, which ranged from black (0.6 cd/m^2^) through to white (69.2 cd/m^2^) in steps of near 2.5 cd/m^2^. Luminance and chromaticity coordinates were verified with a CRS ColourCal (Cambridge Research Systems, Rochester, UK). Targets were greyscale photographs of an object. There were 30 object categories (e.g., ‘frog’, ‘lemon’, see S1 Table for full list) and five different examples of each category creating 150 different stimuli (mean width: 2°; mean height: 4°). The stimuli came from several online databases: The Bank of Standardized Stimuli [50], Object Categories [51], and Shutterstock Inc. (www.shutterstock.com). All visual stimuli were prepared with Matlab (The MathWorks Inc., 2012) with the Psychophysics toolbox [52]. Audio cues (volume-normalised) were recordings of a native British English speaker speaking the same 30 object category labels as the photographs (e.g., “frog”) plus one sample of white noise generated using Audacity software (www.audacityteam.org) and of a duration of 400 ms. Audio cues were presented via HD201 headphones (Sennheiser electronic GmbH & Co. KG, Wedemark, Germany). Participants responded during the trials with a custom-made button box.

#### 1.1.3. Procedure

**Fig 1:**
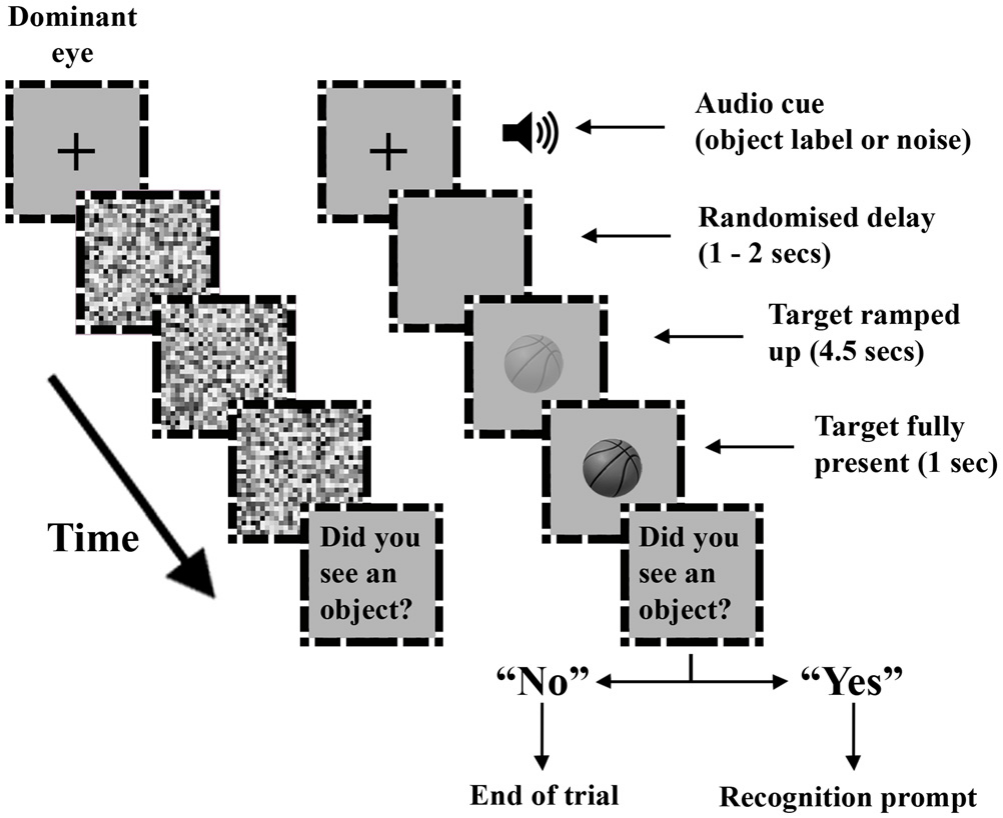
Experiment 1 task procedure for object-present trials. An audio cue (either an object label or white noise) was presented over headphones before the dominant eye was presented with flashing (10 Hz) square random noise patterns. On half the trials the audio cue was an object label and on the other half the audio cue was white noise. Targets were greyscale photos presented to the other eye.

We measured participants’ ability to detect target objects suppressed from visual awareness. For a graphic of the procedure see Fig 1. There were 300 randomised trials of which half contained a target. Therefore, each of the 150 stimuli was displayed once. At the start of every trial an audio cue was played. For object-present trials, half (i.e., 75 trials) contained an object label (e.g., “basketball”) and the other half contained white noise. For object-present trials preceded by an object label, the label matched the object 80% of the time (congruent condition) and did not match it 20% of the time (incongruent condition). This ensured that a label was predictive of the suppressed object. For object-absent trials, 75 were preceded by white noise and 75 by a randomised object label. After the audio cue the fixation crosses were removed and the masking stimuli began to flash to the dominant eye. On object-absent trials only the background grey was displayed inside the box presented to the non-dominant eye and the masking stimuli continued to flash to the dominant eye until the end of the trial (or until the participant made a response). On object-present trials a randomised delay ranging 1–2 seconds was implemented after the audio cue before the target was ramped up to its maximum value (i.e., no transparency) over a period of 4.5 seconds. Once fully revealed and if no detection-response had been made the target remained on screen for a further second. If a detection response was made during the masking period, the masking stimuli and target were removed. If the detection response was “Yes”, a written recognition-probe was then displayed asking whether the object seen matched the audio label that was presented at the start of the trial (e.g., “Was the object a basketball?”). On noise trials the recognition probe was randomised. On trials where no detection response was made before the end of the masking period the target and masking stimuli were replaced with a written prompt asking whether an object had been seen.

### 1.2. Results

We investigated the factor of audio cue (congruent label, incongruent label, and white noise) across four measures of detection performance for all three experiments. Firstly, we analysed reaction times for object-present trials where a response was made before the trial timed out. We excluded trials from this analysis which timed out to avoid contaminating this measure with data recorded after a participant had read and comprehended the written prompt “Did you see an object?” Secondly, we analysed hit rates (accuracy) for all object-present trials. Thirdly, we calculated detection sensitivity (*d’*). Finally, we analysed false alarm rates for object-absent trials. The three dependent variables of reaction time, hit rate, and detection sensitivity were each analysed separately with a repeated-measures ANOVA (subject-based). False alarm rates were analysed as a paired-samples *t*-test to compare this measure on trials containing white noise versus trials containing an object label. Consistent with [35], we also carried out an item-based analysis of hit rates; this additional analysis is commonly included to evaluate the results in language-based experiments [53]. Where appropriate, Greenhouse-Geisser corrections were applied for violations of sphericity. Significant main effects were followed up with planned comparisons comprising paired-samples *t*-tests and alpha was adjusted by the Bonferroni correction for multiple comparisons (α = .05/3).

**Fig 2:**
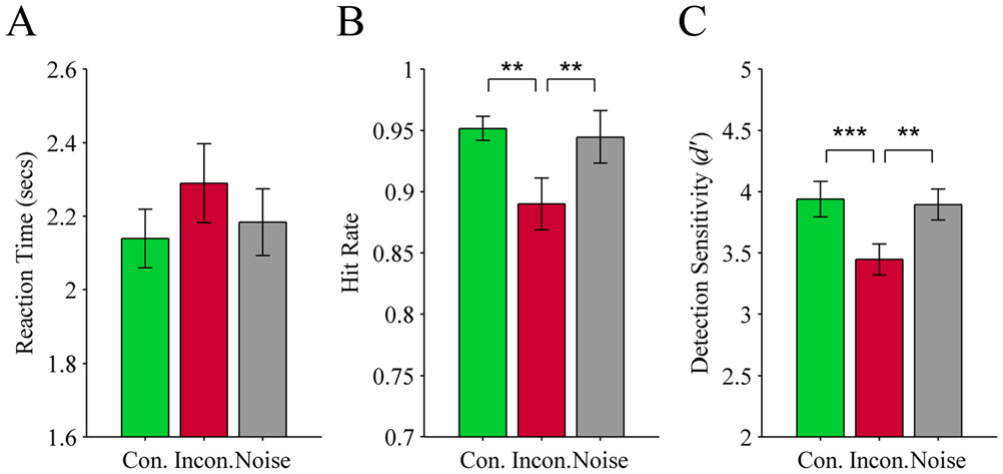
Results from Experiment 1 (object targets preceded by an object label or noise). Three measures of performance for detecting greyscale objects suppressed from visual awareness through CFS: (A) Reaction time, (B) Hit rate, and (C) Detection sensitivity (*d’*). The objects were preceded by one of three types of audio cue: Con. (congruent object label; green), Incon. (incongruent object label; red), or Noise (grey). Error bars show ± 1 SEM. ** *p* < .01; *** *p* < .001.

**Reaction time:** There was no significant effect of audio cue on the speed that participants correctly saw a target, *F*(2, 38) = 2.7, *p* = .079, see Fig 2a.

**Hit rate:** There was a significant effect of audio cue on hit rates, *F*(1.4, 27.1) = 10.7, *p* = .001 (subject-based). Planned comparisons revealed that performance on incongruent trials (*M* = 0.89; *SEM* = 0.02) was significantly poorer than congruent trials (*M* = 0.95; *SEM* = 0.01; *p* = .006) and on trials preceded by noise (*M* = 0.95; *SEM* = 0.01; *p* = .01). Performance on congruent and noise trials did not differ significantly (*p* = .99), see Fig 2b. An item-based analysis of the data reflected the same outcome, *F*(1.0, 134.4) = 6.0, *p* = .016. In this analysis we included the additional factor of stimulus size in pixels (small versus large; based on a median split) as an additional between-subjects factor. This was carried out to investigate whether hit rates were more or less affected by a preceding audio cue depending on the strength of the bottom-up signal, i.e., the size of the stimuli. This would be reflected by a significant audio cue by stimulus size interaction on detection performance, however the interaction was not significant, *F*(2, 264) = 0.0, *p* = .98, and neither was the main effect of stimulus size, *F*(1,132) = 0.4, *p* = .54. Likewise, we found that participants’ hit rates did not correlate with the size of the stimuli (for noise trials *r* = -.04, *p* = .67).

**Detection sensitivity:** There was a significant effect of audio cue on *d’*, *F*(2,38) = 9.9, *p* < .001. Planned comparisons revealed the same trend as hit rates; performance on incongruent trials (*M* = 3.45; *SEM* = 0.13) was significantly poorer than on congruent trials (*M* = 3.94; *SEM* = 0.15, *p* < .001) and noise trials (*M* = 3.90; *SEM* = 0.12; *p* = .008), but congruent and noise trials did not differ significantly (*p* = .99), see Fig 2c.

**False alarm rate:** The number of false alarms did not significantly differ for trials preceded by a verbal label (*M* = 0.17; *SEM* = 0.004) compared to noise (*M* = 0.18; *SEM* = 0.004), *t*(19) = -0.16, *p* = .87.

### 1.3. Discussion

The type of audio cue presented prior to a suppressed object affected participants’ performance at detecting the object. This supports the findings of [35,40], who likewise report that audio cues influence the subsequent detection of suppressed objects. There were however subtle differences between the results of the two studies. Similarly to [35] we found that hit rates and detection sensitivity were poorer on trials when an object label did not match a hidden object (incongruent trials) compared to baseline noise trials and when they did match (congruent trials). However, we did not replicate the finding from [35] that detection is improved when a label matches a hidden object (congruent trials) relative to baseline trials. That is to say that we do not find that appropriate language boosts detection, but rather find that inappropriate language impedes detection. Further, we did not find a significant effect of audio cue on reaction times. Interestingly, [40] report an effect of verbal cues on participants’ reaction times to suppressed stimuli, however they report a different trend to [35]. Unlike [35] who found congruent cues facilitated detection, [40] report a pattern consistent with that observed in our Experiment 1, namely, that incongruent cues significantly impede detection, whereas congruent cues have no effect compared to baseline noise trials. Thus it appears that there is some disagreement in the literature about the direction of the effect of verbal cues on detection performance. The possible neural mechanisms underpinning these different outcomes (boosting performance on congruent trials versus hindering performance on incongruent trials), are addressed in the General Discussion.

## Experiment 2: Colour Targets and Colour Terms

In Experiment 1, we found that object labels affected the detection of greyscale objects hidden from visual awareness, with incongruent labels impeding detection of the objects. A central question here is whether this effect occurs exclusively for objects preceded by object labels, or whether this effect generalises to other types of stimuli and language-stimulus associations. This question is important because if the finding represents a general rule of the effect of language on vision then it should generalise across different types of visual stimulus. In Experiment 2 we tackled this question by suppressing patches of colour, which were preceded by a colour term or by audio noise. We specifically chose to use colour stimuli because of the extensive debate about the stage of colour processing that colour terms might affect. For example, as outlined in the introduction, there is some evidence that colour terms can affect early, perceptual stages of visual processing [18], yet others have failed to replicate these early effects, finding an influence of colour terms only at later, so-called ‘post-perceptual’ stages of visual processing [34]. In the current experiment, by suppressing colour patches through CFS, the colour stimuli are first presented outside of awareness. Consequently, if colour terms do affect early, unconscious processes [18], colour terms would be expected to affect the detection of colour stimuli suppressed with CFS. Conversely, if colour terms only affect later post-perceptual processes [34], they might not be expected to affect the detection of visually suppressed coloured stimuli.

### 2.1. Methods

#### 2.1.1. Participants

Twenty British English speakers took part (15 female; mean age = 21.3; *SD* = 1.5; Range = 20–26). One participant was excluded for failing to follow the task instructions. None of the participants took part in Experiment 1. Participants were recruited from the University of Sussex. All participants were screened for colour vision deficiencies using the same tests as Experiment 1. Observers were naive to the purpose of the study, provided written informed consent and their time was reimbursed with money or research credits. The study was approved by the Cluster-based Ethics Research Committee of Psychology and Life Sciences at the University of Sussex.

#### 2.1.2. Stimuli and set up

The only difference in hardware and set up from that of Experiment 1 was the use of a mirror stereoscope with OptoSigma mirrors (OptoSigma, Santa Ana, USA). While both stereoscopes ultimately provide the same perceptual experience the OptoSigma equipment was preferred for its greater flexibility in adjusting the dichoptic presentation of stimuli. All aspects of the dichoptic stimuli were the same as Experiment 1 except that targets were circular patches of colour (2° × 2°), the edges of which were blurred with a Gaussian filter (σ = 0.08°). The target colours were the eight chromatic basic colours proposed by [54]: Red, orange, yellow, green, blue, purple, pink, and brown, plus white and black. The chromaticity coordinates of these stimuli were taken from data from [55], who investigated English speakers’ focal colours using the Munsell colour system. Focal colours are the best example of a colour when the three dimensions of colour (lightness, saturation and hue) can be varied [56,57]. We converted these Munsell values into CIE *x*,*y*,*Y* values based on an assumed white point of illuminant C with a luminance of 40 cd/m^2^. For those focal colours located outside of the monitor’s gamut, saturation was reduced until the colour could be displayed (see Table 1 for final CIE *x*,*y*,*Y* values). The audio colour cues were likewise these 10 colour terms recorded from a native British English speaker (e.g., “red”). The same background grey and software was used as Experiment 1.

**Table 1:** Chromaticity coordinates (CIE1931, *x*,*y*,*Y*) of colour patch targets from Experiment 2.

Focal colour name	*x*	*y*	*Y*
Red	0.579	0.330	4.8
Orange	0.549	0.395	12.0
Yellow	0.451	0.475	27.4
Green	0.258	0.399	4.8
Blue	0.179	0.189	7.9
Purple	0.278	0.181	4.8
Pink	0.372	0.296	17.2
Brown	0.434	0.402	2.6
White	0.310	0.316	69.2
Black	0.310	0.316	0.58

#### 2.1.3. Procedure

Participants completed 320 trials. Like Experiment 1, half the trials contained targets and half of these colour-present trials were preceded by an audio cue of which 80% matched the colour shown (congruent trials). All other aspects of the procedure were the same as Experiment 1, except that verbal cues were colour terms, and targets were circular patches of colour.

### 2.2. Results

**Fig 3:**
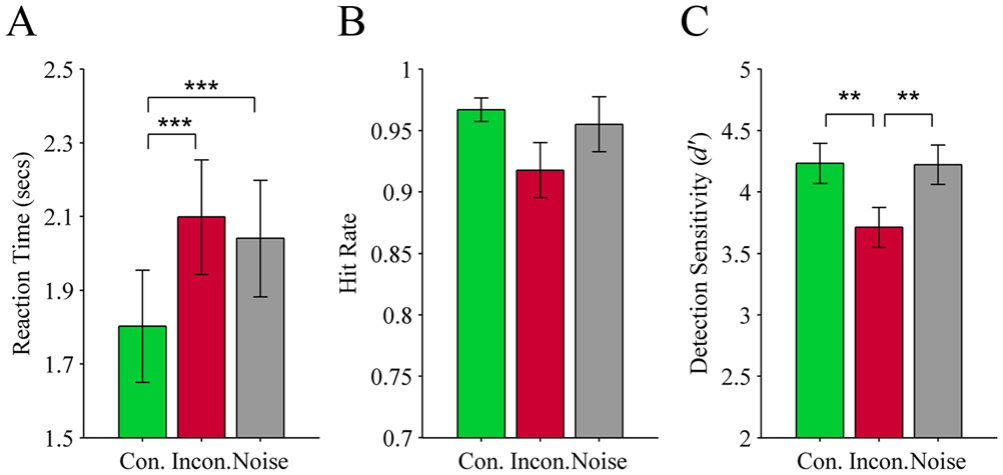
Results from Experiment 2 (colour targets preceded by a colour term or noise). Three measures of performance for detecting circular colour patches suppressed from visual awareness through CFS: (A) Reaction time, (B) Hit rate, and (C) Detection sensitivity (*d’*). The colour patches were preceded by one of three types of audio cue: Con. (congruent colour term; green), Incon. (incongruent colour term; red), or Noise (grey). Error bars show ± 1 SEM. ** *p* < .01; *** *p* < .001.

**Reaction time:** The speed that participants correctly saw a target was significantly affected by the type of audio cue preceding the target, *F*(2, 36) = 19.6, *p* < .001. On congruent trials (*M* = 1.80 seconds; *SEM* = 0.15) performance was significantly faster than both incongruent trials (*M* = 2.10 seconds; *SEM* = 0.16; *p* < .001) and noise trials (*M* = 2.04 seconds; *SEM* = 0.16; *p* < .001). Although reaction times were slowest for incongruent trials, this did not differ significantly from noise trials (*p* = .57), see Fig 3a.

**Hit rate:** There was a significant main effect of audio cue on hit rates, *F*(1.2, 21.1) = 4.3, *p* = .046 (subject-based). Hit rates were highest on congruent trials (*M* = 0.97; *SEM* = 0.010) and lowest on incongruent trials (*M* = 0.92; *SEM* = 0.022), though this difference did not survive Bonferroni correction. Performance on noise trials fell in the middle (*M* = 0.96; *SEM* = 0.014), see Fig 3b. The item-based analysis mirrored this finding for the main effect of audio cue, *F*(2,16) = 4.7, *p* = .025. In the item-based analysis we included the additional factor of colour difference with two levels (small versus large difference). Colour difference values for each stimulus were calculated as the perceptual distance of the colours from the background grey in the perceptually uniform CIELUV colour space and are denoted by ΔE*_uv_. These were categorised as small or large differences based on a median split. The inclusion of colour difference permitted us to assess whether stimuli with a weaker versus stronger bottom-up signal (inferred from the colour difference) were differentially affected by a preceding colour term. There was neither a significant audio cue x colour difference interaction, *F*(2,16) = 2.1, *p* = .154, nor a significant main effect of colour difference, *F*(1,8) = 1.4, *p* = .27.

**Detection sensitivity:** There was a significant main effect of audio cue on *d’*, *F*(1.5, 27.6) = 11.0, *p* = .001. As in Experiment 1, *d’* was significantly lowest on incongruent trials (*M* = 3.71; *SEM* = 0.16) compared to congruent trials (*M* = 4.23; *SEM* = 0.16; *p* = .001) and noise trials (*M* = 4.22; *SEM* = 0.16; *p* = .003), but congruent and noise trials did not differ significantly (*p* = .90), see Fig 3c. The effectiveness of the CFS technique to mask the stimuli was not found to differ significantly between the colour patches used in Experiment 2 (mean *d’*: 4.1) and objects used in Experiment 1 (mean *d’*: 3.8), *t*(37) = -1.6, *p* = .11.

**False alarm rate:** There was no significant difference in false alarm rates on trials with a colour term (*M* = 0.19; *SEM* = 0.011) compared to noise trials (*M* = 0.10; *SEM* = 0.004), *t*(18) = 1.03, *p* = .32.

### 2.3. Discussion

Hearing colour terms affected the subsequent detection of coloured patches suppressed from visual awareness with CFS. Specifically, when a colour term matched the colour of a suppressed target, participants responded significantly faster than when the colour term did not match the target or when just audio noise was heard. This effect was considerable; participants responded around 300 ms faster on congruent trials compared to incongruent trials, and around 250 ms faster compared to noise trials. For both detection sensitivity and hit rates there was a different pattern of results compared to reaction times. Here, the pattern was similar to Experiment 1, whereby colour terms hindered performance on these measures (significantly so for detection sensitivity) when a colour term did not match the colour of a suppressed target (incongruent trials) compared to congruent and noise trials. Overall, these data suggest that the strength of association between a colour term and a colour is strong enough to affect the detection of a coloured patch that has been suppressed from visual awareness through CFS. We next investigated whether a similar effect of language could be found for weaker associations between a verbal label and object by assessing whether verbal colour labels also affect the detection of suppressed images of colour-associated objects presented in greyscale.

## Experiment 3: Object Targets and Colour Terms

The first two experiments found that a verbal cue, which either matched or did not match the object identity of a target (Experiment 1) or the colour of a target (Experiment 2), significantly affected performance at detecting the target when it was suppressed from visual awareness. In Experiment 3 targets were suppressed greyscale object images preceded by the auditory presentation of a colour term (or noise). Consequently, this was an indirect cue to a suppressed stimulus. We used the same object stimuli as Experiment 1, but here the objects were preceded by colour terms in place of the object names. The objects were all associated with a single particular colour. For example, carrots are typically orange; frogs are typically green, and so forth. Importantly, as in Experiment 1, the objects were greyscale and therefore contained no chromatic information. As such, the colour term was a cue to a physical characteristic associated with the hidden object, rather than a direct cue towards the identity of the object. On congruent trials the colour term matched the object’s stereotypical colour and on incongruent trials it did not. We used objects and colour terms because there is evidence that suggests that there is a strong association between colour and object identity. For example, it has been shown that people’s memory of an object’s stereotypical colour modulates the actual appearance of the object [58,59]. Further, a neural representation of object memory colour has also been found in activity in area V1 suggesting that prior knowledge of an object’s colour influences early visual processing [60]. This experiment consequently aimed to further investigate whether the association between colour and object identity is strong enough for colour terms to affect the detection of object images when they are suppressed from visual awareness. As the label-target association relates to a characteristic of the target image rather than its identity, this manipulation further tests the limits of the effect found in Experiments 1 and 2.

### 3.1. Methods

#### 3.1.1. Participants

Twenty British English speakers took part (17 female; mean age = 20.7; *SD* = 0.7; Range = 19–22). None of the participants took part in Experiment 1 or 2. Three participants did not follow the task instructions and were consequently excluded from subsequent analysis. Participants were recruited from the University of Sussex. All participants were screened for colour vision deficiencies using the same tests as Experiment 1. Observers were naive to the purpose of the study, provided written informed consent and their time was reimbursed with money or research credits. The study was approved by the Cluster-based Ethics Research Committee of Psychology and Life Sciences at the University of Sussex.

#### 3.1.2. Stimuli and set up

The visual stimuli were the same greyscale objects used in Experiment 1. These objects were selected a priori because they were each strongly associated with a single, particular colour (verified through pilot testing). Recall the stimuli in Experiment 1 comprised 30 object categories (e.g., ‘banana’) and there were five different examples of each category (150 stimuli in total). Out of the 30 categories, five were associated with green (frog, lettuce, peapod, broccoli, and pear). There were therefore 25 individual green-associated stimuli. The same was the case for the colours red, orange, yellow, pink, and brown (see S1 Table for full list of objects and colours). Note that targets were greyscale and contained no chromatic information. Audio cues were the names of each of the six above mentioned colour terms. The hardware and all other aspects of the visual stimuli were the same as Experiment 1.

#### 3.1.3. Procedure

Participants completed 300 trials. As in Experiment 1, half the trials contained objects of which half were preceded by an audio cue and half by white noise. On congruent trials the audio colour cue matched the object’s characteristic colour (e.g., “orange” for basketball; “yellow” for banana etc.). On incongruent trials the colour cue did not match the object’s characteristic colour. As with the prior experiments, on object-present trials preceded by a colour cue, 80% were congruent, 20% were incongruent, and on object-absent trials the audio cue was randomised (half were white noise).

### 3.2. Results

**Fig 4:**
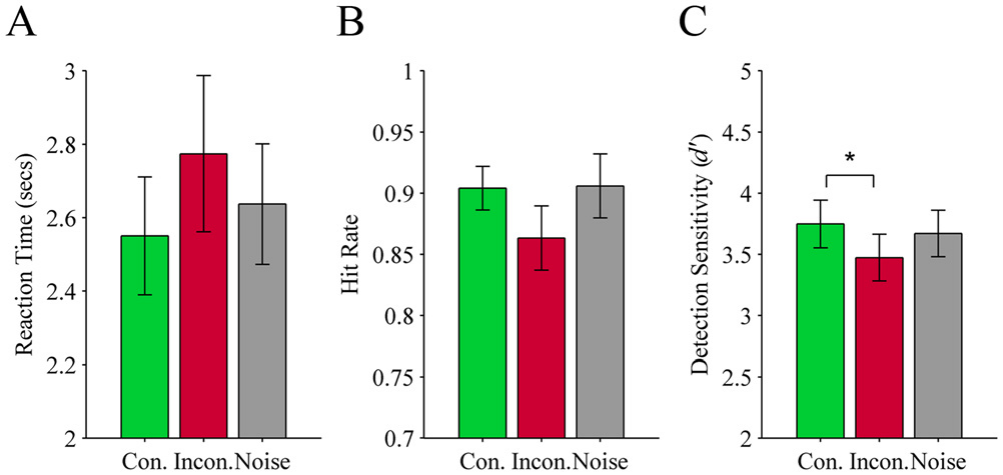
Results from Experiment 3 (object targets preceded by a colour term or noise). Three measures of performance for detecting greyscale objects suppressed from visual awareness using CFS: (A) Reaction time, (B) Hit rate, and (C) Detection sensitivity (*d’*). The objects were each associated with a single colour (e.g., a banana is associated with yellow) and were preceded by one of three types of audio cue: Con. (congruent colour term; green), Incon. (incongruent colour term; red), or Noise (grey). Error bars show ± 1 SEM. * *p* < .05.

**Reaction time:** There was no significant main effect of audio cue on reaction times, *F*(1.2, 18.5) = 2.10, *p* = .163, see Fig 4a.

**Hit rate:** There was a marginal main effect of audio cue on hit rates, *F*(2, 32) = 3.12, *p* = .058 (subject-based). The trend was similar to the prior experiments in that hit rates were lowest on incongruent trials (*M* = 0.86; *SEM* = 0.026) compared to congruent (*M* = 0.90; *SEM* = 0.018) and noise trials (*M* = 0.91; *SEM* = 0.026), see Fig 4b. As for Experiments 1 and 2, we ran an item-based analysis on this data. The outcome indicated that audio cue did not significantly affect hit rates, *F*(1.0, 137.7) = 2.43, *p* = .12. We again included the factor of stimulus size in pixels (small versus large) to assess whether hit rate was more or less affected by a verbal cue depending on stimulus size. There was no significant audio cue x stimulus size interaction (*F* < 1). There was however a main effect of stimulus size, *F*(1, 135) = 5.3, *p* = .02, with hit rates significantly lower for small (*M* = 0.87; *SEM* = 0.16) compared to large stimuli (*M* = 0.92; *SEM* = 0.16) based on a median split. There was also a significant correlation between stimulus size and hit rates for noise trials, *r* = .32, *p* < .001, as well as congruent trials, *r* = .34, *p* < .001, but not for incongruent trials, *r* = .09, *p* = .34.

**Detection sensitivity:** There was a significant main effect of audio cue on detection sensitivity, *F*(2, 32) = 4.56, *p* = .018. Planned comparisons revealed *d’* was significantly higher on congruent trials (*M* = 3.75; *SEM* = 0.20) compared to incongruent trials (*M* = 3.47; *SEM* = 0.19; *p* = .015), but not noise trials (*M* = 3.67; *SEM* = 0.19; *p* = .343). The difference in *d’* between noise and incongruent trials did not reach significance (*p* = .056), see Fig 4c.

**False alarm rate:** There was no significant difference in false alarm rates on verbal label trials (*M* = 0.02; *SEM* = 0.018) compared to noise trials (*M* = 0.02; *SEM* = 0.020), *t*(16) = -1.00, *p* = .33.

### 3.3. Discussion

In the previous experiments, observers were given a verbal cue that either matched or did not match the identity of a hidden target. In Experiment 3 we instead investigated whether the association between colour terms and object identity is strong enough to affect the detection of hidden objects. The verbal cue was a colour term that either matched or did not match the typical colour associated with a hidden object. Importantly, target objects were greyscale, therefore the colour term was an indirect cue to a physical characteristic associated with the object, rather than a direct cue. We found that, despite no chromatic information being available to assist detection, colour terms affected the detection of hidden, greyscale objects. Specifically, detection sensitivity was significantly worse on incongruent compared to congruent trials. For example, hearing the cue “yellow” impeded the detection of an object not typically associated with that colour relative to the detection of an object associated with that colour. There was however, no significant difference in the effect of verbal label on detection relative to just hearing noise. A similar, though marginal effect was found for hit rates, and as was the case for Experiment 1 we did not find that the verbal cues significantly affected reaction times to hidden targets. Given the lack of significant effects for two of the measures, and the lack of significant difference between the verbal labels and noise, the evidence for the effect of terms on the detection of suppressed stimuli appears less convincing for colour term-object associations than for when terms directly relate to the identity of an object.

One possibility for the weaker effect in Experiment 3 may be a weaker association between colour labels and objects compared to colour labels and colours or object labels and objects. For example, while a carrot may typically be associated with the colour orange it may also be associated with the green colour of its foliage. A further point to consider is that while the item-based analysis revealed no overall significant effect of verbal labels on hit rates, unlike Experiments 1 and 2 we found that the stimulus strength, here indexed by the image size, was positively associated with hit rates. Although this finding might be expected (more stimulus strength or bottom-up signal should assist detection), it is unclear why this pattern was observed in Experiment 3 where colour labels were used and not in Experiment 1 where object labels were used for the same stimuli. These data suggest that the physical characteristics of a hidden stimulus may play a greater role in determining the detection of the stimulus when a preceding verbal label is an indirect clue to the hidden stimulus. However, without further investigation it is unclear why this was the case for congruent and noise trials, but not for incongruent trials.

## General Discussion

Previous studies have found that hearing an object label affects detection of an object suppressed from visual awareness through CFS [35,40]. Importantly, both these studies provide evidence that an object label both improved detection when the label matched the object, and inhibited detection when it did not. In Experiment 1 we partially replicated this effect. Unlike these previous studies, we did not find that detection is improved by a congruent object label, but we did find that detection was worse when a label did not match a suppressed image. However, in Experiment 2, in which colour patches were suppressed and preceded by a colour term or noise, like [35,40] we found that these linguistic cues can both facilitate detection (reaction times were significantly faster on congruent trials compared to the other conditions), as well as hinder detection (*d’* was significantly lower on incongruent trials compared to the other conditions). One possibility may arise from the colour stimuli used in Experiment 2 each being presented 16 times, whereas the stimuli in Experiments 1 and 3 were presented just once. This may have resulted in greater predictive power of the colour terms which in turn increased the power of the effect. In Experiment 3, we found a weaker effect. For both reaction times and hit rates we found that verbal labels comprising the typical colour of a suppressed, greyscale object had no significant effect on detecting the objects compared to baseline. Detection sensitivity was however affected and here the trend was for colour terms to impede detection on incongruent trials compared to congruent trials.

In the present study we extend the finding that verbal labels affect the detection of suppressed objects by demonstrating that this effect generalises to colour stimuli and colour-associated objects preceded by a colour term. This suggests that the association between a colour term and a colour is strong enough to affect whether and when a coloured patch will be perceived when it is suppressed from awareness through CFS. There has been much debate about the stage of colour processing that colour terms might affect. Some data suggest colour terms affect early stages of visual processing [18], while some indicate this effect resides in later, post-perceptual stages of processing [34]. It has been proposed that CFS disrupts processing prior to semantic analysis (e.g., [37]; for a discussion see below). If so, the present study provides further support for colour terms affecting earlier stages of processing (e.g., [18]). We also show that the association between objects and colour terms is strong enough to affect detection of suppressed colour-associated objects. This supports prior findings that memory of an object’s typical colour is enough to affect the appearance of an object [58,59], and may be related to the finding of colour-associated neural activity in area V1 that has been shown to be elicited in response to greyscale colour-associated objects [60].

The difference between boosting detection on congruent trials and inhibiting detection on incongruent trials has important theoretical implications because it likely speaks to the mechanism underlying how language can affect the detection of a suppressed stimulus. One idea is that language modulates bottom-up processing in the visual system by affecting neural signals associated with processing a stimulus [3,21]. However, the question is how is this achieved? One possibility for the finding that language can improve detection, as observed in Experiment 2 and in the studies by [35,40], is that language may advance the specific signal associated with processing the suppressed stimulus compared to background neural noise in the visual processing system. According to [61], this is consistent with some electrode-recording experiments, which show that activity from higher-level areas can reduce (and refine) activity in early visual processing areas [62]. A candidate suggested by [61] for this process is population encoding (e.g., [63]).

In the current study we find stronger evidence that language can obstruct detection, rather than facilitate detection. One possible account of this finding is that an incongruent label adds further noise to the visual system, which consequently hinders detection. If the higher-level visual areas in extrastriate cortex represent hypotheses about visual stimuli, which are tested against information in earlier stages of vision such as area V1 (e.g., [64]), ambiguity in early visual signals will not be resolved as successfully on incongruent trials compared to congruent trials because the hypothesis is incorrect (the verbal label does not match the stimulus). In this view, feed-forward pathways, which contain both the neural signal and the residual error signal between predictions, may contain greater error in the signal (e.g., [65]).

If language does feedback to earlier stages of visual processing [3,35], this requires specification of the higher and lower-level visual areas that are responsible for this process and the manner in which this process occurs. Lupyan and Ward [35] identify the occipito-temporal cortex, a region with an established role in object recognition, as a candidate for the higher-level cortical area mediating the effects of language on the detection of suppressed stimuli. The account holds that recognition of the object name drives a top-down process providing input to this region. That input sets up a cognitive expectation for visual input arriving from lower-level visual regions that matches the representation elicited by the word. CFS has been shown to modulate the cortical activity at those low-level cortical regions; fMRI reveals reliable reductions in responses observed in V1 for CFS suppressed targets [66]. Research exploring the process by which CFS achieves this suppressing effect has identified two key mechanisms; attentional modulation (e.g.,[67]) and divisive normalisation (e.g., [68]). Stimulus driven attentional modulation operates by virtue of the salient mask causing attentional selection for the location and orientation of that visual competitor. This has the effect of reducing the gain for neural responses to the stimulus and increasing the gain for the neural responses to the mask. Divisive normalisation is a process whereby the responses of neurons responding to some target are divided by the responses to a wider (normalising) population which include the distractor or mask. The presence of the highly activating mask in the normalising population has the effect of reducing relative activation from the target. In combination these two influences suppress the target in a similar fashion to reducing contrast.

While potential candidates for the higher-level representation of language that drives the effects reported in the present study have been discussed, it remains unclear which lower-level stage(s) of visual processing language may penetrate. [61] suggest that higher-level areas could feedback to area V1, while attention has been shown to modulate activity even earlier, in the lateral geniculate nucleus [69]. Concerning our finding that colour terms affect colour detection, a key question for future research will be to clarify whether the low-level areas involved are the same as for suppressed objects, or whether feedback occurs to alternative areas. For example, it is possible that the lower-level area could be one that is highly specialised for the processing of colour, such as area V4 [29,70,71].

An essential foundation for the conclusions drawn by [35] is that CFS disrupts processing prior to semantic analysis and that the effect of verbal labels found on detection must occur in earlier, lower-level stages of visual processing. But is this necessarily the case? There are a number of studies that support this view. They fail to find an observable effect on behaviour from a suppressed prime, which suggests that semantic processing is inhibited for suppressed stimuli. For example, no priming effects were reported for suppressed objects [38], and binocular rivalry, more generally, has been reported to obstruct semantic processing of words [39]. This has also been demonstrated by measuring electrophysiological activity, whereby the N400 component, which is associated with language processing (for a review see, [72]), was absent for words suppressed through CFS [37]. However, there are several contrary findings that show a priming effect (and presumably semantic processing) for stimuli suppressed using CFS. This has been shown for suppressed words [73], as well as numbers [74]. Another finding to consider is that of [75], who showed that novel word combinations break from CFS suppression faster if they contain semantic violations compared to when they do not. This conflicts with a principal finding of the present study, namely that stimuli with a weaker association (i.e., the incongruent condition) are less easily detected. Clearly, the manner in which suppressed stimuli reach perception is not yet fully understood, but importantly these conflicting findings may suggest that it is not a strictly feed-forward process (e.g., [5,6]) and that multiple mechanisms may play a role in this processes. Unravelling the behavioural contexts that affect perception of suppressed stimuli will be key to furthering our understanding of how suppressed stimuli are processed, and may in turn shed light on the way our perceptual system operates as a whole.

We do not yet have a precise method to study the effect of language on perception. It has been argued that the effect of language on the detection of stimuli suppressed by CFS indicates that language affects early stages rather than post-perceptual stages of visual processing [35,40]. In the present study we sought to add to this debate by testing the generality of this effect. While we replicated and extended the effect, albeit with some differences, we are cautious about interpreting this as evidence that language affects early stages of processing during CFS. Given that participants were required to respond once a suppressed stimulus was perceived, we cannot rule out the possibility that verbal labels may act at the stage of decision rather than detection itself [15,16]. For example [15] propose that visual adaptation to implied motion may best be attributed to a decision-level bias, rather than earlier-occurring sensory-level changes in the visual system, because an adaptation-induced bias in reported stimulus direction only occurred when the participants’ task involved a directional judgement. That influence disappeared on a non-directional task. Similarly [16] report that task strategy may account for effects found on tasks probing visual acuity. Further research into the neural locus of this effect will likely shed light on this question. Transcranial magnetic stimulation has previously been used successfully to disrupt processing in areas, such as the inferior occipito-temporal cortex, which are relevant to this discussion[76,77]. This could prove to be a useful tool for investigating whether this area plays a role in the effects reported here, as proposed by [35]. Likewise, neuroimaging studies will be crucial for investigating whether language (via verbal cues) is fed back to earlier stages of vision. While fMRI data suggests this is the case for attention on visual search [78], it remains to be clarified whether it is also the case for language.

## Supporting Information

S1 Table Category listing for stimuli in Experiments 1 and 3. (DOCX)

## References

[1] Lee P. The Whorf Theory Complex: A Critical Reconstruction. Philadelphia, PA: John Benjamins Pub Co; 1996.

[2] Lucy JA. Linguistic relativity. Annu Rev Anthropol. 1997; 291–312.

[3] Lupyan G. Linguistically Modulated Perception and Cognition: The Label-Feedback Hypothesis. Front Psychol. 2012;3: 1–13. 10.3389/fpsyg.2012.00054 22408629 PMC3297074

[4] Pinker S. The Language Instinct: The New Science of Language and Mind. New York: HarperCollins; 1995.

[5] Seidenberg MS, McClelland JL. A distributed developmental model of word recognition and naming. Psychol Rev. 1989;96: 523–568. 10.1037/0033-295X.96.4.523 2798649

[6] Stone GO, Van Orden GC. Strategic control of processing in word recognition. J Exp Psychol Hum Percept Perform. 1993;19: 744 8409857 10.1037//0096-1523.19.4.744

[7] Kinchla RA, Wolfe JM. The order of visual processing:“Top-down,”“bottom-up,” or “middle-out” Percept Psychophys. 1979;25: 225–231. 461079 10.3758/bf03202991

[8] McClelland JL, Rumelhart DE. An interactive activation model of context effects in letter perception: Part 1. An account of basic findings. Psychol Rev. 1981;88: 375–407. 10.1037/0033-295X.88.5.375 7058229

[9] Mesulam MM. From sensation to cognition. Brain. 1998;121: 1013–1052. 9648540 10.1093/brain/121.6.1013

[10] Stone GO, Vanhoy M, Van Orden GC. Perception is a two-way street: Feedforward and feedback phonology in visual word recognition. J Mem Lang. 1997;36: 337–359.

[11] Lupyan G. The conceptual grouping effect: Categories matter (and named categories matter more). Cognition. 2008;108: 566–577. 10.1016/j.cognition.2008.03.009 18448087

[12] Kranjec A, Lupyan G, Chatterjee A. Categorical Biases in Perceiving Spatial Relations. Bremner A, editor. PLoS ONE. 2014;9: e98604 10.1371/journal.pone.0098604 24870560 PMC4037194

[13] Winawer J, Witthoft N, Frank MC, Wu L, Wade AR, Boroditsky L. Russian blues reveal effects of language on color discrimination. Proc Natl Acad Sci. 2007;104: 7780–7785. 10.1073/pnas.0701644104 17470790 PMC1876524

[14] Roberson D, Davidoff J, Davies IRL, Shapiro LR. Color categories: Evidence for the cultural relativity hypothesis. Cognit Psychol. 2005;50: 378–411. 10.1016/j.cogpsych.2004.10.001 15893525

[15] Mather G, Sharman RJ. Decision-level adaptation in motion perception. R Soc Open Sci. 2015;2: 150418 10.1098/rsos.150418 27019726 PMC4807448

[16] Morgan M, Dillenburger B, Raphael S, Solomon JA. Observers can voluntarily shift their psychometric functions without losing sensitivity. Atten Percept Psychophys. 2012;74: 185–193. 10.3758/s13414-011-0222-7 22033949 PMC3264850

[17] Luck SJ. An Introduction to the Event-Related Potential Technique. Cambridge, Mass: MIT Press; 2005.

[18] Thierry G, Athanasopoulos P, Wiggett A, Dering B, Kuipers J-R. Unconscious effects of language-specific terminology on preattentive color perception. Proc Natl Acad Sci. 2009;106: 4567–4570. 10.1073/pnas.0811155106 19240215 PMC2657373

[19] Androulaki A, Gômez-Pestaña N, Mitsakis C, Jover JL, Coventry K, Davies IRL. Basic colour terms in Modern Greek: Twelve terms including two blues. J Greek Linguist. 2006;45: 3–47.

[20] Clifford A, Holmes A, Davies IRL, Franklin A. Color categories affect pre-attentive color perception. Biol Psychol. 2010;85: 275–282. 10.1016/j.biopsycho.2010.07.014 20674661

[21] Boutonnet B, Lupyan G. Words Jump-Start Vision: A Label Advantage in Object Recognition. J Neurosci. 2015;35: 9329–9335. 10.1523/JNEUROSCI.5111-14.2015 26109657 PMC6605198

[22] Di Russo F, Martínez A, Sereno MI, Pitzalis S, Hillyard SA. Cortical sources of the early components of the visual evoked potential. Hum Brain Mapp. 2002;15: 95–111. 10.1002/hbm.10010 11835601 PMC6871868

[23] Hopf J-M, Vogel E, Woodman G, Heinze H-J, Luck SJ. Localizing Visual Discrimination Processes in Time. J Neurophysiol. 2002;88: 2088–2095. 12364530 10.1152/jn.2002.88.4.2088

[24] Johannes S, Münte TF, Heinze HJ, Mangun GR. Luminance and spatial attention effects on early visual processing. Cogn Brain Res. 1995;2: 189–205. 10.1016/0926-6410(95)90008-X 7580401

[25] Donchin E, Coles MG. Is the P300 component a manifestation of context updating? Behav Brain Sci. 1988;11: 357–374.

[26] Patel SH, Azzam PN. Characterization of N200 and P300: selected studies of the event-related potential. Int J Med Sci. 2005;2: 147–54. 16239953 10.7150/ijms.2.147 PMC1252727

[27] Brouwer GJ, Heeger DJ. Categorical Clustering of the Neural Representation of Color. J Neurosci. 2013;33: 15454–15465. 10.1523/JNEUROSCI.2472-13.2013 24068814 PMC3782623

[28] Zeki SM. A century of cerebral achromatopsia. Brain. 1990;113: 1721–1771. 10.1093/brain/113.6.1721 2276043

[29] Zeki SM. Functional organization of a visual area in the posterior bank of the superior temporal sulcus of the rhesus monkey. J Physiol. 1974;236: 549–573. 4207129 10.1113/jphysiol.1974.sp010452 PMC1350849

[30] Bird CM, Berens SC, Horner AJ, Franklin A. Categorical encoding of color in the brain. Proc Natl Acad Sci. 2014;111: 4590–4595. 10.1073/pnas.1315275111 24591602 PMC3970503

[31] Persichetti AS, Thompson-Schill SL, Butt OH, Brainard DH, Aguirre GK. Functional magnetic resonance imaging adaptation reveals a noncategorical representation of hue in early visual cortex. J Vis. 2015;15: 1–19. 10.1167/15.6.18 PMC4461891 26024465

[32] Pulvermüller F, Shtyrov Y. Language outside the focus of attention: The mismatch negativity as a tool for studying higher cognitive processes. Prog Neurobiol. 2006;79: 49–71. 10.1016/j.pneurobio.2006.04.004 16814448

[33] Clifford A, Franklin A, Holmes A, Drivonikou VG, Özgen E, Davies IRL. Neural correlates of acquired color category effects. Brain Cogn. 2012;80: 126–143. 10.1016/j.bandc.2012.04.011 22722021

[34] He X, Witzel C, Forder L, Clifford A, Franklin A. Color categories only affect post-perceptual processes when same-and different-category colors are equally discriminable. J Opt Soc Am A. 2014;31: A322–A331. 10.1364/JOSAA.31.00A322 24695189

[35] Lupyan G, Ward EJ. Language can boost otherwise unseen objects into visual awareness. Proc Natl Acad Sci. 2013;110: 14196–14201. 10.1073/pnas.1303312110 23940323 PMC3761589

[36] Tsuchiya N, Koch C. Continuous flash suppression reduces negative afterimages. Nat Neurosci. 2005;8: 1096–1101. 10.1038/nn1500 15995700

[37] Kang M-S, Blake R, Woodman GF. Semantic Analysis Does Not Occur in the Absence of Awareness Induced by Interocular Suppression. J Neurosci. 2011;31: 13535–13545. 10.1523/JNEUROSCI.1691-11.2011 21940445 PMC3209531

[38] Cave CB, Blake R, McNamara TP. Binocular rivalry disrupts visual priming. Psychol Sci. 1998;9: 299–302.

[39] Zimba LD, Blake R. Binocular rivalry and semantic processing: Out of sight, out of mind. J Exp Psychol Hum Percept Perform. 1983;9: 807–815. 6227690 10.1037//0096-1523.9.5.807

[40] Sun Y, Cai Y, Lu S. Hemispheric asymmetry in the influence of language on visual perception. Conscious Cogn. 2015;34: 16–27. 10.1016/j.concog.2015.03.004 25840357

[41] Corballis MC. The Lopsided Ape: Evolution of the Generative Mind. New York: Oxford University Press; 1993.

[42] Hellige JB. Hemispheric Asymmetry: What’s Right and What’s Left. Harvard University Press; 1993.

[43] Drivonikou GV, Kay P, Regier T, Ivry RB, Gilbert AL, Franklin A, et al Further evidence that Whorfian effects are stronger in the right visual field than the left. Proc Natl Acad Sci. 2007;104: 1097–1102. 10.1073/pnas.0610132104 17213312 PMC1783370

[44] Gilbert AL, Regier T, Kay P, Ivry RB. Whorf hypothesis is supported in the right visual field but not the left. Proc Natl Acad Sci U S A. 2006;103: 489–494. 10.1073/pnas.0509868103 16387848 PMC1326182

[45] Becker E, Karnath H-O. Incidence of Visual Extinction After Left Versus Right Hemisphere Stroke. Stroke. 2007;38: 3172–3174. 10.1161/STROKEAHA.107.489096 17962601

[46] Ringman JM, Saver JL, Woolson RF, Clarke WR, Adams HP. Frequency, risk factors, anatomy, and course of unilateral neglect in an acute stroke cohort. Neurology. 2004;63: 468–474. 15304577 10.1212/01.wnl.0000133011.10689.ce

[47] Arnold DH, Law P, Wallis TSA. Binocular switch suppression: A new method for persistently rendering the visible “invisible.” Vision Res. 2008;48: 994–1001. 10.1016/j.visres.2008.01.020 18329066

[48] Ishihara S. Ishihara test for colour-blindness. Tokyo: Kanehara & Co. Ltd; 1987.

[49] Fletcher R. The City University Colour Vision Test. 2nd ed London: Keeler; 1980.

[50] Brodeur MB, Dionne-Dostie E, Montreuil T, Lepage M. The Bank of Standardized Stimuli (BOSS), a New Set of 480 Normative Photos of Objects to Be Used as Visual Stimuli in Cognitive Research. Op de Beeck HP, editor. PLoS ONE. 2010;5: e10773 10.1371/journal.pone.0010773 20532245 PMC2879426

[51] Konkle T, Brady TF, Alvarez GA, Oliva A. Scene Memory Is More Detailed Than You Think: The Role of Categories in Visual Long-Term Memory. Psychol Sci. 2010;21: 1551–1556. 10.1177/0956797610385359 20921574 PMC3397240

[52] Brainard DH. The psychophysics toolbox. Spat Vis. 1997;10: 433–436. 9176952

[53] Walker ECT. Performing item-and subject-based analyses using ALICE. Behav Res Methods Instrum. 1977;9: 3–4.

[54] Berlin B, Kay P. Basic color terms: their universality and evolution. Berkeley: University of California; 1969.

[55] Sturges J, Whitfield TW. Locating basic colours in the Munsell space. Color Res Appl. 1995;20: 364–376. 10.1002/col.5080200605

[56] Miyahara E. Focal colors and unique hues. Percept Mot Skills. 2003;97: 1038–1042. 10.2466/pms.2003.97.3f.1038 15002843 PMC1404500

[57] Kuehni RG. Focal Color Variability and Unique Hue Stimulus Variability. J Cogn Cult. 2005;5: 409–426. 10.1163/156853705774648554

[58] Hansen T, Olkkonen M, Walter S, Gegenfurtner KR. Memory modulates color appearance. Nat Neurosci. 2006;9: 1367–1368. 10.1038/nn1794 17041591

[59] Olkkonen M, Hansen T, Gegenfurtner KR. Color appearance of familiar objects: Effects of object shape, texture, and illumination changes. J Vis. 2008;8: 13–13. 10.1167/8.5.13 18842084

[60] Bannert MM, Bartels A. Decoding the Yellow of a Gray Banana. Curr Biol. 2013;23: 2268–2272. 10.1016/j.cub.2013.09.016 24184103

[61] Kersten D, Yuille A. Bayesian models of object perception. Curr Opin Neurobiol. 2003;13: 150–158. 10.1016/S0959-4388(03)00042-4 12744967

[62] Lee TS, Yang CF, Romero RD, Mumford D. Neural activity in early visual cortex reflects behavioral experience and higher-order perceptual saliency. Nat Neurosci. 2002;5: 589–597. 10.1038/nn860 12021764

[63] Pouget A, Dayan P, Zemel R. Information processing with population codes. Nat Rev Neurosci. 2000;1: 125–132. 11252775 10.1038/35039062

[64] Grill-Spector K, Kourtzi Z, Kanwisher N. The lateral occipital complex and its role in object recognition. Vision Res. 2001;41: 1409–1422. 11322983 10.1016/s0042-6989(01)00073-6

[65] Rao RP, Ballard DH. Predictive coding in the visual cortex: a functional interpretation of some extra-classical receptive-field effects. Nat Neurosci. 1999;2: 79–87. 10195184 10.1038/4580

[66] Yuval-Greenberg S, Heeger DJ. Continuous Flash Suppression Modulates Cortical Activity in Early Visual Cortex. J Neurosci. 2013;33: 9635–9643. 10.1523/JNEUROSCI.4612-12.2013 23739960 PMC3760788

[67] Li H-H, Carrasco M, Heeger DJ. Deconstructing Interocular Suppression: Attention and Divisive Normalization. Bethge M, editor. PLOS Comput Biol. 2015;11: e1004510 10.1371/journal.pcbi.1004510 26517321 PMC4627721

[68] Baker DH, Graf EW. Natural images dominate in binocular rivalry. Proc Natl Acad Sci. 2009;106: 5436–5441. 10.1073/pnas.0812860106 19289828 PMC2663995

[69] O’Connor DH, Fukui MM, Pinsk MA, Kastner S. Attention modulates responses in the human lateral geniculate nucleus. Nat Neurosci. 2002;11: 1203–1209. 10.1038/nn957 12379861

[70] Brouwer GJ, Heeger DJ. Decoding and Reconstructing Color from Responses in Human Visual Cortex. J Neurosci. 2009;29: 13992–14003. 10.1523/JNEUROSCI.3577-09.2009 19890009 PMC2799419

[71] Conway BR, Moeller S, Tsao DY. Specialized Color Modules in Macaque Extrastriate Cortex. Neuron. 2007;56: 560–573. 10.1016/j.neuron.2007.10.008 17988638 PMC8162777

[72] Kutas M, Federmeier KD. Thirty Years and Counting: Finding Meaning in the N400 Component of the Event-Related Brain Potential (ERP). Annu Rev Psychol. 2011;62: 621–647. 10.1146/annurev.psych.093008.131123 20809790 PMC4052444

[73] Costello P, Jiang Y, Baartman B, McGlennen K, He S. Semantic and subword priming during binocular suppression. Conscious Cogn. 2009;18: 375–382. 10.1016/j.concog.2009.02.003 19286396 PMC4521603

[74] Bahrami B, Vetter P, Spolaore E, Pagano S, Butterworth B, Rees G. Unconscious Numerical Priming Despite Interocular Suppression. Psychol Sci. 2010;21: 224–233. 10.1177/0956797609360664 20424051

[75] Sklar AY, Levy N, Goldstein A, Mandel R, Maril A, Hassin RR. Reading and doing arithmetic nonconsciously. Proc Natl Acad Sci. 2012;109: 19614–19619. 10.1073/pnas.1211645109 23150541 PMC3511729

[76] Duncan KJ, Pattamadilok C, Devlin JT. Investigating occipito-temporal contributions to reading with TMS. J Cogn Neurosci. 2010;22: 739–750. 10.1162/jocn.2009.21207 19302001 PMC2827369

[77] Mancini F, Bolognini N, Bricolo E, Vallar G. Cross-modal processing in the occipito-temporal cortex: a TMS Study of the Müller-Lyer illusion. J Cogn Neurosci. 2011;23: 1987–1997. 10.1162/jocn.2010.21561 20807050

[78] Melloni L, van Leeuwen S, Alink A, Muller NG. Interaction between Bottom-up Saliency and Top-down Control: How Saliency Maps Are Created in the Human Brain. Cereb Cortex. 2012;22: 2943–2952. 10.1093/cercor/bhr384 22250291

