# Supplementary material for: Colour Terms Affect Detection of Colour and Colour-Associated Objects Suppressed from Visual Awareness
Source: PLoS One. 2016 Mar 29;11(3):e0152212. doi: 10.1371/journal.pone.0152212 (PMC4811409; doi:10.1371/journal.pone.0152212)
Supplement: S1 Table — (DOCX) [file pone.0152212.s001.docx]

Colour Terms Affect Detection of Colour and Colour-associated Objects Suppressed from Visual Awareness

PLOS ONE (2015)

Lewis Forder^1*^, Olivia Taylor^1^, Helen Mankin^1^, Ryan B. Scott^2^, Anna Franklin^1^

**1** The Sussex Colour Group, School of Psychology, University of Sussex, Falmer, Brighton, United Kingdom, **2** School of Psychology, University of Sussex, Falmer, Brighton, United Kingdom

| **S1 Table. Category listing for stimuli in Experiments 1 and 3.** | | | |
| --- | --- | --- | --- |
| Banana | (yellow) | Pea pod | (green) |
| Basketball | (orange) | Pear | (green) |
| Broccoli | (green) | Pig | (pink) |
| Carrot | (orange) | Piggy Bank | (pink) |
| Chocolate | (brown) | Pine cone | (brown) |
| Coconut | (brown) | Pineapple ring | (yellow) |
| Corn on cob | (yellow) | Prawn | (pink) |
| Fire engine | (red) | Pumpkin | (orange) |
| Fire extinguisher | (red) | Rubber Duck | (yellow) |
| Flamingo | (pink) | Santa Hat | (red) |
| Ham | (pink) | Strawberry | (red) |
| Frog | (green) | Tiger | (orange) |
| Lemon | (yellow) | Tomato | (red) |
| Lettuce | (green) | Traffic cone | (orange) |
| Moose | (brown) | Violin | (brown) |
| The colour terms in parentheses refer to the pairing between object and colour on congruent trials in Experiment 3. | | | |
